# Environmental Salinity Determines the Specificity and Need for Tat-Dependent Secretion of the YwbN Protein in *Bacillus subtilis*

**DOI:** 10.1371/journal.pone.0018140

**Published:** 2011-03-30

**Authors:** René van der Ploeg, Ulrike Mäder, Georg Homuth, Marc Schaffer, Emma L. Denham, Carmine G. Monteferrante, Marcus Miethke, Mohamed A. Marahiel, Colin R. Harwood, Theresa Winter, Michael Hecker, Haike Antelmann, Jan Maarten van Dijl

**Affiliations:** 1 Department of Medical Microbiology, University Medical Center Groningen and University of Groningen, Groningen, The Netherlands; 2 Interfaculty Institute for Genetics and Functional Genomics, Department for Functional Genomics, Ernst-Moritz-Arndt-University Greifswald, Greifswald, Germany; 3 Department of Chemistry/Biochemistry, Philipps-University Marburg, Marburg, Germany; 4 Centre for Bacterial Cell Biology, Institute of Cell and Molecular Biosciences, Newcastle University, Newcastle upon Tyne, United Kingdom; 5 Institut für Mikrobiologie und Molekularbiologie, Ernst-Moritz-Arndt-Universität Greifswald, Greifswald, Germany; Vrije Universiteit Brussel, Belgium

## Abstract

Twin-arginine protein translocation (Tat) pathways are required for transport of folded proteins across bacterial, archaeal and chloroplast membranes. Recent studies indicate that Tat has evolved into a mainstream pathway for protein secretion in certain halophilic archaea, which thrive in highly saline environments. Here, we investigated the effects of environmental salinity on Tat-dependent protein secretion by the Gram-positive soil bacterium *Bacillus subtilis*, which encounters widely differing salt concentrations in its natural habitats. The results show that environmental salinity determines the specificity and need for Tat-dependent secretion of the Dyp-type peroxidase YwbN in *B. subtilis*. Under high salinity growth conditions, at least three Tat translocase subunits, namely TatAd, TatAy and TatCy, are involved in the secretion of YwbN. Yet, a significant level of Tat-independent YwbN secretion is also observed under these conditions. When *B. subtilis* is grown in medium with 1% NaCl or without NaCl, the secretion of YwbN depends strictly on the previously described “minimal Tat translocase” consisting of the TatAy and TatCy subunits. Notably, in medium without NaCl, both *tatAyCy* and *ywbN* mutants display significantly reduced exponential growth rates and severe cell lysis. This is due to a critical role of secreted YwbN in the acquisition of iron under these conditions. Taken together, our findings show that environmental conditions, such as salinity, can determine the specificity and need for the secretion of a bacterial Tat substrate.

## Introduction

The transport of proteins across biological membranes and their subsequent secretion into external milieus are vital processes for all known microorganisms. These processes depend on the activity of dedicated molecular machines. A first critical step in protein secretion is the passage of transported proteins through the cytoplasmic membrane, which can occur either in an unfolded state *via* the general secretion (Sec) machinery, or in a folded state *via* the twin-arginine translocation (Tat) machinery [1], [2], [3], [4], [5]. Accordingly, the Sec and Tat machines work independently of each other, using distinct mechanisms for protein translocation. Acceptance of a protein by the Sec or Tat complexes is dictated by the presence of an N-terminal signal peptide with or without a selective recognition motif for Tat (*i.e*. the twin-arginine motif) [6], and the folding state of the transported protein [7], [8]. In general, Tat-dependent proteins fold prior to translocation, while the folding of Sec-dependent proteins occurs post-translocationally [7].

Early studies have defined the twin-arginine (RR-) motif in the N-region of signal peptides as S/T-R-R-x-F-L-K [6], [9], [10], [11], [12]. However, this motif is not always strictly conserved in Tat-dependently exported proteins. The observed natural variations, as well as site-directed mutagenesis studies, have defined a more general RR-consensus sequence as R/K-R-x-#-#, where # is a hydrophobic residue [13], [14], [15]. This RR-motif is recognized by the membrane-embedded Tat machinery of which two general types are known. Gram-negative bacteria, like *Escherichia coli*, contain a TatABC-type machinery consisting of three Tat proteins (TatA, TatB and TatC) that are indispensable for translocation [16], [17]. In contrast, most Gram-positive bacteria contain a “minimal” TatAC machinery that lacks the TatB protein [18], [19]. In these minimal TatAC translocases, the role of TatB is fulfilled by a bifunctional TatA protein [20], [21]. The precise mechanism of Tat-dependent protein translocation is currently unknown, but studies in *E. coli* and the thylakoids of plant chloroplasts have shown that RR-signal peptide recognition involves a TatB-TatC complex [22], [23], [24]. Subsequently, TatBC-precursor complexes merge with TatA sub-complexes to facilitate the translocation process in such a way that folded proteins of varying sizes and complexity can pass across the membrane [24], [25].

Notably, some organisms contain multiple Tat translocases [26]. This was shown for the Gram-positive bacterium *Bacillus subtilis*, which contains two minimal TatAC translocases named TatAdCd and TatAyCy that can function independently of each other [20]. The *tatAd-tatCd* and *tatAy-tatCy* genes coding for these translocases are organised in operons at separate genomic loci [18]. In what follows these operons are referred to as *tatAdCd* and *tatAyCy*, respectively. *B. subtilis* contains a third *tatA* gene (*tatAc*) with no demonstrable role in Tat-dependent protein transport [19], [20]. The specificities of the TatAdCd and TatAyCy translocase complexes are non-identical, but overlapping. Only two *B. subtilis* proteins, YwbN and PhoD, have been shown to be secreted in a strictly Tat-dependent manner [19], [20]. YwbN is a member of the family of Dyp-type peroxidases that contain an iron-heme co-factor [27]. This protein is a preferred substrate for the constitutively expressed TatAyCy translocase when cells are grown in standard Luria-Bertani (LB) medium. In contrast, secretion of the phosphodiesterase PhoD is strictly dependent on the TatAdCd translocase. Notably, the *phoD* gene is located upstream of the *tatAd*-*tatCd* genes, and all three genes are induced under phosphate starvation conditions [18].

*In silico* analyses suggested that 69 proteins of *B. subtilis* have signal peptides with potential RR-motifs [19]. However, proteomics and molecular biological analyses revealed that only two of these - YwbN and PhoD – were strictly dependent on a functional Tat translocase for secretion. The signal peptides of the *B. subtilis* QcrA and YkuE proteins were shown to direct Tat-dependent protein secretion in *Streptomyces* [28], but the Tat-dependence of these proteins in *B. subtilis* remains to be shown. Recently, we demonstrated that the esterase LipA can be secreted via both the Sec and Tat pathways of *B. subtilis* [29]. This became evident under conditions of LipA hyperproduction, indicating that an overflow mechanism exists to re-direct LipA from the normally used Sec-dependent export route into the Tat-dependent route. Importantly, if LipA is produced at wild-type levels this protein is secreted Sec-dependently and, in accordance with this view, no Tat-dependent secretion of the LipA protein can be demonstrated in the sequenced *B. subtilis* strain 168 [19], [29]. These observations on LipA secretion suggest that seemingly Sec-dependent proteins can be targeted to the Tat pathway depending on intracellular and perhaps even extracellular conditions. In this context, it is noteworthy that the Tat machinery is extensively used for protein secretion by certain halophilic archaea, such as *Haloferax volcanii* and *Haloarcula hispanica*, indicating that Tat has evolved into a mainstream secretion pathway for some organisms that grow in highly saline milieus [30], [31], [32], [33], [34], [35]. This raised the question as to what extent salinity can impact on Tat-dependent protein secretion by microorganisms, like the soil bacterium *B. subtilis*, that live in ecological niches where the salinity can fluctuate markedly. As a first approach to address this question, we studied the influence of different NaCl concentrations in the growth medium of *B. subtilis* on the Tat-dependent secretion of the YwbN protein. The results show that the NaCl content of the growth medium has a strong impact on the Tat-dependency of YwbN secretion. Remarkably, when cells were grown at high salinity some Tat-independent secretion of YwbN was observed. Moreover, the Tat-dependent secretion of YwbN at high salinity does not only involve the TatAy and TatCy subunits as previously shown, but also the TatAd subunit. If no NaCl is included in the LB medium, only TatAy and TatCy are required for YwbN secretion. Under these conditions TatAy and TatCy are of major importance for growth and cell viability, which relates to an important role of YwbN in iron acquisition.

## Results

### TatAyCy-independent secretion of YwbN at high salinity

**Figure 1:**
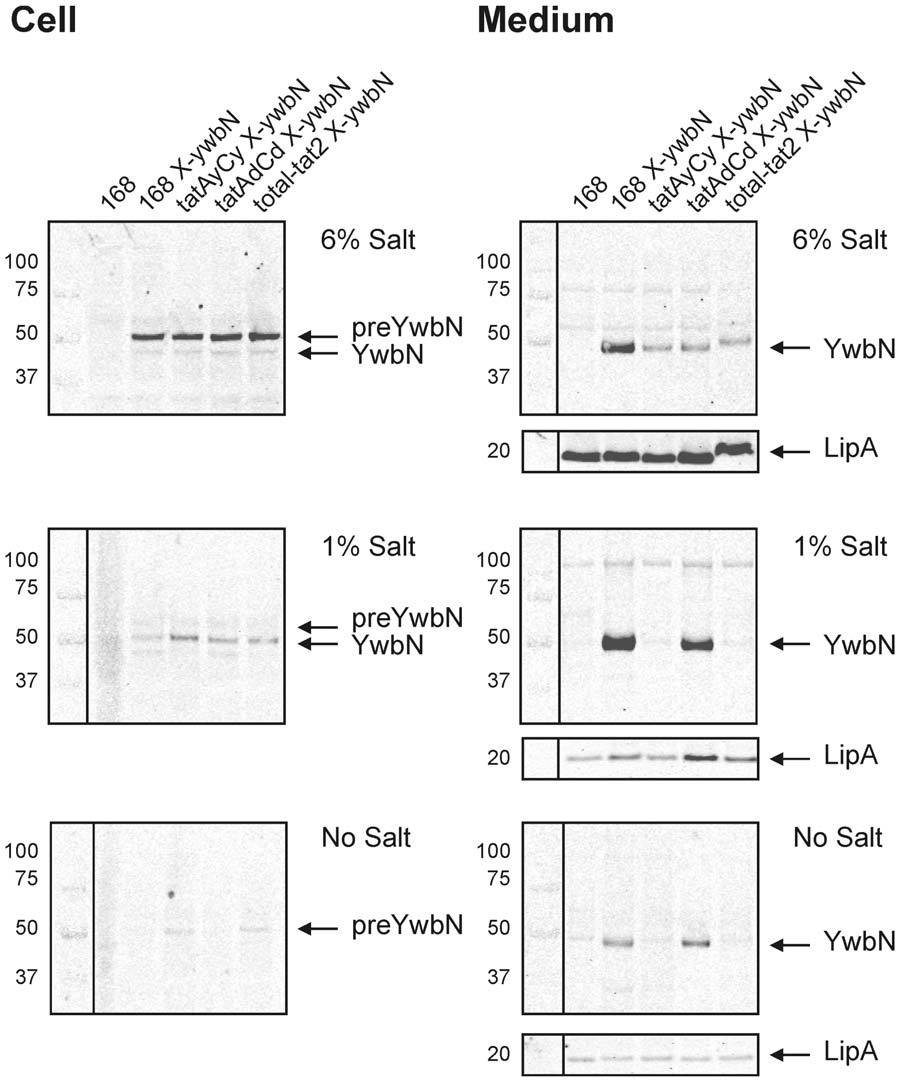
Tat-dependence of YwbN secretion in media with differing salinity. Cell and growth medium fractions of *B. subtilis tat* mutant strains and the parental strain 168 were separated by centrifugation and used for SDS-PAGE, Western blotting and immunodetection of YwbN-Myc and LipA with specific antibodies. From top to bottom the panels show results obtained for cells grown in LB with 6%, 1% or no added NaCl. Protein loading was corrected for OD_600_. The YwbN-Myc (YwbN and preYwbN) and LipA proteins, and Mw markers are indicated. A slight ‘smiling effect' as observed for the YwbN and LipA bands in the growth medium sample of the total-*tat*2 mutant grown in LB with 6% NaCl is due to some residual salt in the sample (see also Figure S2).

To investigate whether high salinity might impact on Tat-dependent secretion, we investigated the secretion of YwbN in LB medium with a final salt concentration of 6%, which is comparable to that of highly saline environments or brine. It should be noted that the ‘standard’ LB medium, as originally defined by Luria and Bertani and used in all our previously published secretion studies, already contains 1% NaCl. Thus, its salinity resembles that of brackish water or the cytoplasm. LB with no added salt would reflect the situation encountered in fresh water. To facilitate YwbN detection, this protein was provided with a C-terminal Myc-epitope, and the corresponding gene construct was expressed ectopically from the xylose-inducible *xylA* promoter. The cassette encoding the *xylA-ywbN-myc* fusion was named X-YwbN (Table 1). When the cells reached an OD_600_ of 2, expression of *ywbN* was induced and growth was continued for 3 hours. Cellular and growth medium fractions were collected and used for SDS-PAGE and Western blotting to monitor YwbN secretion. The results revealed a significant impact of high salinity on the specificity of YwbN secretion (Figure 1, upper panels). Clearly, YwbN secretion was no longer strictly dependent on the *tatAyCy* genes when cells were grown in medium with 6% salt. Although deletion of *tatAyCy* resulted in a strong reduction of the extracellular level of YwbN, the *tatAyCy* mutant nevertheless secreted substantial amounts of this protein. This contrasts markedly with the secretion of YwbN by cells grown in LB with 1% or no salt, which is strictly TatAyCy-dependent (Figure 1, middle and lower panels). Remarkably, at 6% NaCl the secretion of YwbN was also reduced in a *tatAdCd* mutant. This suggested for the first time an involvement of TatAd and/or TatCd in YwbN secretion in a strain in which *tat* gene expression was not genetically engineered. Furthermore, this observation was intriguing because a function for *tatAdCd* had only been demonstrated so far under phosphate starvation conditions [18]. Consistent with the finding that *tatAyCy* or *tatAdCd* mutant strains secreted reduced amounts of mature YwbN, the secretion of YwbN was also strongly reduced in the total-*tat2* mutant strain (Figure 1), which lacks all *B. subtilis tat* genes (Table 1). These data show that YwbN secretion was partially Tat-independent when cells were grown in the presence of 6% salt. To verify that this Tat-independent secretion of YwbN was not due to cell lysis, the locations of the Sec-dependently secreted LipA protein and the cytoplasmic marker protein TrxA were verified by Western blotting. No extracellular TrxA was detected, indicating that the observed Tat-independent secretion of YwbN was not due to cell lysis (Figure S1). Furthermore, secreted LipA was readily detectable (Figure 1), and Sypro Ruby-staining of the respective gels did not reveal any major differences in the total amounts of protein secreted by the parental and *tat* mutant strains grown in LB with 6% salt (Figure S2). Taken together, these findings show that the TatAyCy translocase is not exclusively involved in YwbN secretion under high salinity growth conditions, and that TatAd and/or TatCd are also involved in this process.

**Table 1:** Strains and Plasmids used in this study.

Plasmids	Relevant properties	Reference
pGDL48	Contains multiple cloning site to place genes under the control of the erythromycin promoter; 6.8 kb; Ap^R^; Km^R^	[54]
pCAd	pGDL48 containing the *tatAd* gene; 7.0 kb; Ap^R^; Km^R^	[36]
pCCd	pGDL48 containing the *tatCd* gene; 7.5 kb; Ap^R^; Km^R^	[36]
pCACd	pGDL48 containing the *tatAd*-*tatCd* operon; 7.7 kb; Ap^R^; Km^R^	[36]
pCAy	pGDL48 containing the *tatAy* gene; 7.0 kb; Ap^R^; Km^R^	[20]
pCCy	pGDL48 containing the *tatCy* gene; 7.5 kb; Ap^R^; Km^R^	[20]
pCACy	pGDL48 containing the *tatAy-tatCy* operon; 7.7 kb; Ap^R^; Km^R^	[20]
pXTc	Expression vector with the xylose-inducible *xylA* promoter; Ap^R^; Tc^R^	[55]
pXTc-ywbN-myc	pXTc with the *ywbN-myc* gene; results in the production of YwbN with a C-terminal Myc tag	This study
***B. subtilis***		
168	*trpC2*	[56]
*tatAy*	*trpC2*; *tatAy*::Em; Em^R^	[20]
*tatCy*	*trpC2*; *tatCy*::Sp; Sp^R^	[18]
*tatAyCy*	*trpC2*; *tatAy*-*tatCy*::Sp; Sp^R^	[19]
*tatCd*	*trpC2*; *tatCd*::Km; Km^R^	[18]
*tatAdCd*	*trpC2*; *tatAd*-*tatCd*::Km; Km^R^;	[20]
*tatAy*-*tatAd*	*trpC2*; *tatAy*::Em; Em^R^ *; tatAd*::Km; Km^R^	[20]
*tatCy*-*tatCd*	*trpC2*; *tatCy*::Sp; Sp^R^; *tatCd*::Km; Km^R^	
total-*tat*	*trpC2, tatAc*::Em; Em^R^ *; tatAy-tatCy*::Sp; Sp^R^ *; tatAd-tatCd*::Cm; Cm^R^	[19]
total-*tat_2_*	*trpC2*; *tatAd*-*tatCd*::Km; Km^R^; *tatAy*-*tatCy*::Sp; Sp^R^; *tatAc*::Em; Em^R^	[20]
168 *X-ywbN*	*trpC2; amyE*::*xylA-ywbN-myc*; Cm^R^	[19]
*tatAyCy X-ywbN*	*trpC2*; *tatAy*-*tatCy*::Sp; Sp^R^; *amyE*::*xylA-ywbN-myc*; Cm^R^	[19]
*tatAdCd X-ywbN*	*trpC2*; *tatAd*-*tatCd*::Km; Km^R^; *amyE*::*xylA-ywbN-myc*; Cm^R^	[19]
*tatAdCd X-ywbN_2_*	*trpC2*; *tatAd*-*tatCd*::Cm; Cm^R^; *amyE*::*xylA-ywbN-myc*; Tc^R^	This study
total-*tat_2_ X-ywbN*	*trpC2*; *tatAd*-*tatCd*::Km; Km^R^; *tatAy*-*tatCy*::Sp; Sp^R^; *tatAc*::Em; Em^R^; *amyE*::*xylA-ywbN-myc*; Cm^R^	[19]
*ywbL*	*trpC2, ywbL::*pMutin2::Em; Em^R^ (BFA3211)	[57]
*ywbM*	*trpC2, ywbM::*pMutin2::Em; Em^R^ (BFA3212)	[57]
*ywbN*	*trpC2, ywbN::*pMutin2::Em; Em^R^ (BFA3213)	[57]
*ywbN* X-*ywbN*	*trpC2, ywbN::*pMutin2::Em; Em^R^ *amyE::xylA-ywbN-myc:* Cm^R^	This study

It should be noted that the cellular amounts of YwbN-Myc decreased when the salt concentration in the growth medium was reduced (Figure 1). It is currently not known why this occurs, but it may relate to the xylose-induced expression of the *xylA*-*ywbN*-*myc* cassette. Furthermore, we also observed that the total yields of secreted LipA became lower when the NaCl concentration in the medium was reduced (Figure 1). This decrease seems specific for LipA as the total amounts of secreted proteins were not substantially affected by differing salt concentrations (Figure S2). Again, this may relate to variation in gene expression under the different conditions, but it is also conceivable that salt has an impact on certain stages in the LipA secretion process.

### TatAd, TatAy and TatCy are required for efficient YwbN secretion at high salinity

**Figure 2:**
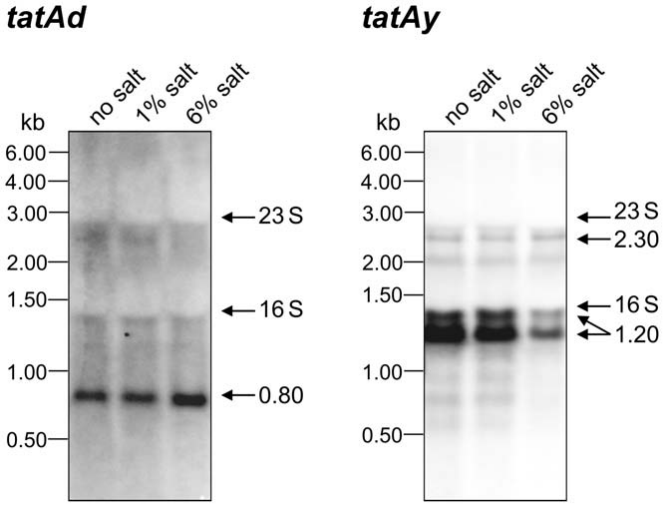
Northern blotting analysis of *tatAd* and *tatAy* transcription in cells grown in LB media of differing salinity. RNA isolation and Northern blotting were performed as described in the Materials and Methods section. For each sample, 5 µg of RNA per lane were loaded as indicated. Molecular markers and the positions of the 16S and 23S rRNA are indicated.

Recent studies by Eijlander *et al*. [36] indicated that engineered *tatAdCd* expression was able to compensate for the absence of the *tatAyCy* genes in the secretion of YwbN. We therefore investigated the levels of *tatAdCd-* and *tatAyCy-* specific transcripts in cells grown in LB media with varying NaCl concentrations by Northern blotting. Interestingly, we identified a 0.8 kb *tatAd*-specific transcript that was detectable at comparable amounts in cells grown in the presence of 1% NaCl or without NaCl (Figure 2). When cells were grown in LB with 6% NaCl, the level of this *tatAd* transcript was elevated. Most likely, it corresponds to the complete *tatAd* gene and part of the *tatCd* gene (data not shown). Conversely, the levels of a *tatAyCy*-specific transcript of ∼1.2 kb showed a clear decrease in cells grown in medium with 6% salt compared to cells grown in medium with 1% salt or no salt. The same is true for some *tatAyCy*-specific read-through transcripts that accumulate in the zone of the rRNA bands, where the electrophoretic separation of mRNAs is somewhat impaired. The ∼1.2 kb transcript and the read-through transcripts originate from a promoter immediately upstream of *tatAy* ([37]; data not shown). The levels of a less abundant ∼2.3 kb transcript corresponding to *moaC*, *rex*, *tatAy* and *tatCy* were not affected by growth medium salinity (Figure 2). These findings imply that the relative levels of TatAd and TatAyCy production can differ in cells grown in LB media of high or low salinity, which may explain the altered Tat component specificity observed for YwbN secretion in medium with 6% salt as shown in Figure 1. Notably, the finding that there was no *tatCd* transcript detectable when cells were grown in LB with 6% salt indicated that TatCd has no, or only a very minor role in YwbN secretion during growth at high salinity.

**Figure 3:**
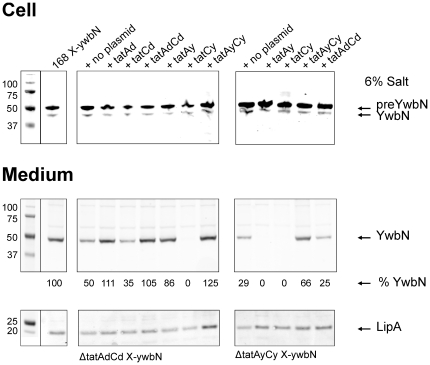
Complementation analysis of *tatAdCd* or *tatAyCy* mutant strains grown at high salinity. Growth medium and cell fractions of *B. subtilis tat* mutant strains and the parental strain 168 grown in LB with 6% added NaCl were separated and used for SDS-PAGE, Western blotting and immunodetection of YwbN-Myc and LipA using specific antibodies. The *tatAdCd* or *tatAyCy* mutant strains (marked at the bottom of the Figure) were complemented with plasmid-borne *tat* genes as indicated at the top of the Figure. The plasmids used for this purpose were pCAd, pCCd, pCACd, pCAy, pCCy, pCACy (see Table 1). Note that the *tatAdCd* mutant strains contained an X-*ywbN* cassette in which the original Cm^R^ marker had been replaced by a Tc^R^ marker (*tatAdCd* X-*ywbN*_2_), whereas the 168 strain and the *tatAyCy* mutant strains contained the previously published X-*ywbN* cassette (see Table 1). The YwbN-Myc (YwbN and preYwbN) and LipA proteins, and Mw markers are indicated.

Consistent with the view that TatAd has a role in YwbN secretion when cells are grown in LB medium with 6% NaCl, the plasmid-directed expression of *tatAd* was sufficient for full restoration of the secretion of YwbN by *tatAdCd* mutant cells under these conditions (Figure 3, compare the first two lanes for the *tatAdCd* mutant strain with the control). Furthermore, YwbN secretion under these conditions was also fully restored by the expression of plasmid-borne *tatAdCd* or *tatAyCy* genes. Conversely, no restoration of YwbN secretion was observed in the *tatAdCd* mutant upon expression of a plasmid-borne *tatCd* gene alone, and YwbN secretion by the *tatAdCd* mutant was even completely suppressed by the presence of a plasmid-borne copy of *tatCy*. The reduced secretion of YwbN by a *tatAyCy* mutant grown in LB with 6% NaCl was complemented by plasmid-borne *tatAyCy*, but it was completely inhibited by the presence of plasmid-borne copies of either *tatAy* or *tatCy* alone (Figure 3, rightside panels). Lastly, expression of plasmid-borne *tatAdCd* genes in the *tatAyCy* mutant did not restore secretion of YwbN, which contrasts with the afore-mentioned findings by Eijlander *et al*. [36]. Taken together, these observations show that TatAd, TatAy and TatCy are involved in the Tat-dependent secretion of YwbN by cells growing in LB with 6% NaCl. This is reminiscent of the situation in Gram-negative bacteria that contain a three-component TatABC translocase. Furthermore, the results indicate that a separate expression of *tatAy* or *tatCy* can strongly interfere with YwbN secretion by cells grown at high salinity.

### TatAyCy is required for optimal growth of B. subtilis and entry into stationary phase at low salinity

**Figure 4:**
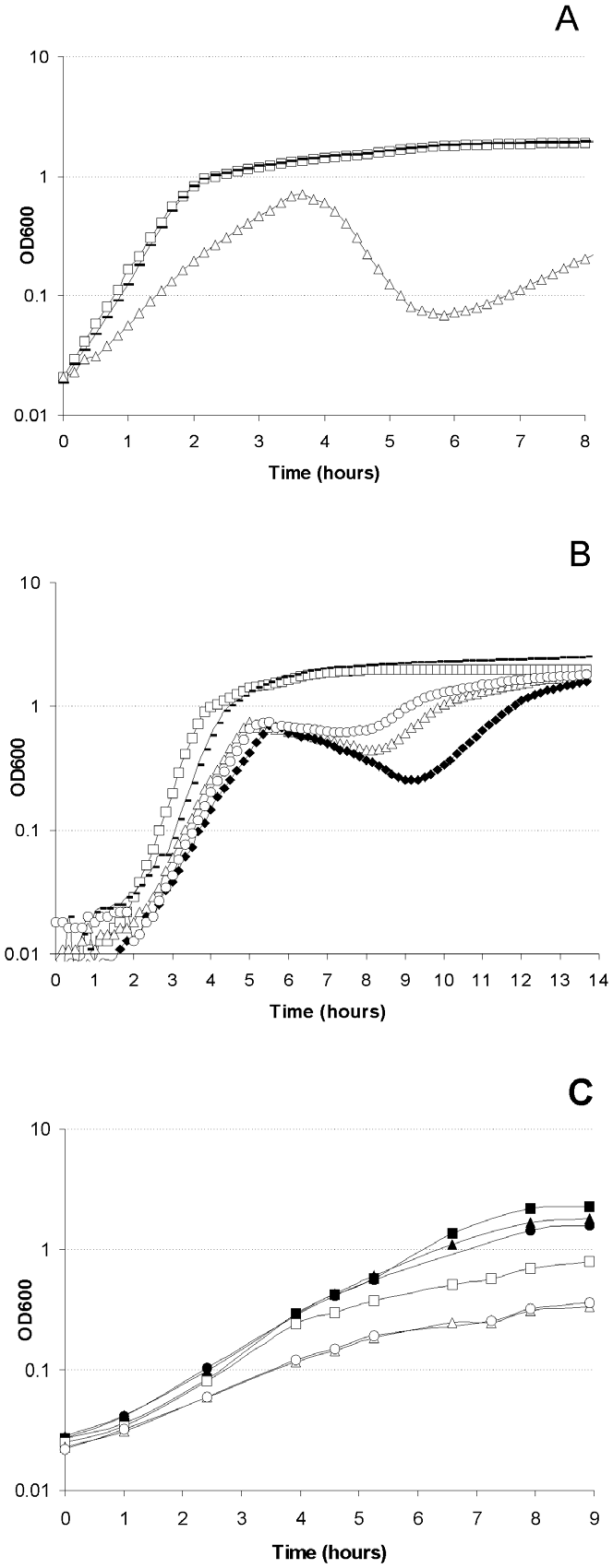
Growth phenotypes of *tatAyCy* and *ywbLMN* mutant strains at low salinity. **A**. Growth of the *B. subtilis* strains *tatAdCd* (filled rectangles), *tatAyCy* (open triangles), and 168 (open squares) in LB medium without NaCl. **B**. Growth of *B. subtilis* strains *ywbN* (open circles), *ywbN* X*ywbN* plus xylose (closed rectangles), *ywbL* (closed diamonds), *tatAyCy* (open triangles), and 168 (open squares) in LB medium without NaCl. **C**. Growth of the *B. subtilis* strains total-*tat* (triangles), total-*tat2* (circles) and 168 (squares) in BOC medium: open symbols, no added iron; closed symbols, addition of 10 µM FeSO_4_.

The experiments to monitor Tat-dependent secretion of YwbN by the *tatAdCd*, *tatAyCy* or total-*tat* mutant strains cultivated in LB medium without added salt revealed an unexpectedly strong growth phenotype for mutant strains lacking the *tatAy* and/or *tatCy* genes (Figure 4A and Figure S3). Compared to the parental strain 168 and *tatAdCd* mutant strains, the *tatAyCy* and total-*tat* mutant strains showed significantly reduced growth rates during the exponential growth phase. Most strikingly, the *tatAyCy* and total-*tat* mutants exhibited a lower growth yield as compared to the *tatAyCy* proficient strains and they were even unable to enter directly into a stationary phase of growth. Instead, mutants lacking *tatAyCy* displayed a severe lysis phenotype as reflected by a significant drop in the OD_600_ of the culture. Interestingly, the surviving *tatAyCy* mutant cells resumed (or continued) growth about 1.5 hours after the onset of cell lysis, but at a lower rate than during the initial exponential growth. This growth phenotype could be reversed by complementation of the chromosomal *tatAyCy* mutations with plasmid-borne copies of the *tatAyCy* genes (Figure S3, panel B). Specifically, the growth defect of cells lacking *tatAy* and *tatCy* at low salinity was only reversed when both the *tatAy* and *tatCy* genes were present on the complementing plasmid. Since *tatAyCy* mutant cells that had recovered from the lysis phase showed the same phenotype upon re-cultivation under low salinity conditions, it seems that the cells that resumed growth had adapted to these conditions rather than expressing a suppressor mutation. Importantly, the observed phenotype revealed that *B. subtilis* needs to have an active TatAyCy translocase for optimal growth and entry into stationary phase at low salinity.

### TatAyCy-dependent secretion of YwbN has an important role in iron acquisition

To test whether the known TatAyCy substrate YwbN was implicated in the growth kinetics observed at low salinity, experiments were performed with a *ywbN* mutant. Indeed, the *ywbN* mutant strain displayed a similar growth phenotype as strains lacking *tatAyCy* in the absence of added salt (Figure 4, panels A and B; Figure S3, panel C), while its growth phenotype was normal in the presence of 1% salt (Figure S3, panel F). Furthermore, the growth phenotype of the *ywbN* mutant strain was fully complemented by xylose-induced ectopic expression of a copy of *ywbN-Myc* that was integrated into the chromosomal *amyE* locus (Figure S3, panel C). Consistent with these findings, the secretion of Myc-tagged YwbN was strictly dependent on TatAyCy under conditions of low salinity (Figure 1, lower panels). Since *ywbN* is part of a well-conserved cluster of three genes of which the two other genes, *ywbL* and *ywbM*, have been implicated in iron uptake [20], [38], [39], we also investigated whether *ywbL* or *ywbM* mutant strains have a growth defect at low salinity. Indeed, this was the case (Figure 4B; Figure S3, panel C), suggesting that YwbL (a homologue of known iron permeases) and YwbM (a lipoprotein), as well as TatAyCy-dependently secreted YwbN could be required for the uptake of sufficient amounts of iron to sustain growth under conditions of low salinity. To test this idea, all growth experiments at low salinity were repeated in the presence of 10 µM FeCl_3_ (Figure S3, panel D) or FeSO_4_ (Figure S3, panel E). The results showed that addition of 10 µM Fe^2+^ to the low salinity LB medium strongly stimulated the growth of the *tatAyCy*, *ywbL*, *ywbM*, or *ywbN* mutants to levels that were indistinguishable from growth in the presence of 1% salt (Figure S3, compare panels E and F). Importantly, the addition of FeSO_4_ to *tatAyCy* or total-*tat* mutant cells growing exponentially in LB without salt completely prevented the occurrence of the lysis phase (Figure S4). This implies that the resumed growth after the lysis phase is most likely due to the release of iron by the lysed cells that can then be used by the surviving cells to resume or continue growth. The addition of 10 µM Fe^3+^ to the low salinity medium had only a moderately stimulating effect on the growth of the *tatAyCy*, *ywbM* or *ywbN* mutant strains, while Fe^3+^ had barely any growth-promoting effect on the *ywbL* mutant strain (Figure S3, panel D). Limited rescue of the mutants by addition of Fe^3+^ is consistent with the previously reported lack of Fe^3+^-specific bacillibactin-dependent iron acquisition in the *B. subtilis* 168 strain [40], while excess of Fe^2+^ is directed towards low affinity uptake by divalent cation importers [41], [42]. The results imply that YwbL is the decisive Fe^3+^ permease for elemental iron uptake as previously suggested [43], and that the *B. subtilis* TatAyCy translocase and its substrate YwbN are of pivotal importance for the acquisition of iron during growth at low salinity. Since this suggests a possible general role for the *B. subtilis* Tat pathway in growth under iron-limited conditions, we tested the growth of two independently constructed *B. subtilis* total-*tat* mutants in synthetic iron-limited BOC medium. The results in Figure 4C show that, indeed, the growth rates of total-*tat* mutants in BOC medium were significantly lower than that of the parental strain 168. This growth defect was largely relieved by the addition of 10 µM Fe^2+^ to the medium (Figure 4C), showing that the *B. subtilis* Tat pathway is required for optimal growth under iron-limited conditions.

## Discussion

In the present studies we have addressed the question as to whether the salinity of the growth medium is a determinant in Tat-dependent secretion of the YwbN protein by *B. subtilis*. The results show that this is indeed the case. Intriguingly, high growth medium salinity had a strong impact on the specificity of the Tat pathway of *B. subtilis*, as shown by the role of the TatAd component in YwbN secretion. The observation that, at high salinity, TatAd was involved in YwbN secretion to a similar extent as TatAyCy implies that the TatAd, TatAy and TatCy subunits of *B. subtilis* can cooperate under these conditions. This contrasts strongly with the previously demonstrated exclusive dependence of YwbN secretion on the TatAyCy translocase, when cells were grown in LB with 1% salt [20]. It was recently reported that YwbN can be secreted in a TatAdCd-dependent manner by cells growing in LB medium with 1% NaCl. However, this only occurs when the TatAdCd translocase is produced ectopically from plasmid-borne *tatAdCd* genes [36]. In this somewhat artificial situation, TatAdCd was able to sustain YwbN secretion in the absence of TatAyCy, which is clearly not the case for the parental 168 strain growing at high salinity (Figure 3, last lane), where cooperation between TatAd, TatAy and TatCy appears to be necessary for optimal YwbN secretion via Tat. Interestingly, our Northern blotting analyses indicate that *B. subtilis* is able to fine-tune the levels of TatAd, TatAy and TatCy in cells grown in high salinity medium through an as yet unidentified transcriptional regulatory mechanism. The present findings thus show that the TatAd subunit is not only involved in Tat-dependent protein secretion upon phosphate starvation, but also when high levels of salt are present in the growth medium. To date, it is not known whether the observed cooperativity between TatAd, TatAy and TatCy is triggered directly by high levels of salt (*e.g.* high ionic strength), or by the altered expression levels of *tatAd* and *tatAyCy*.

Cells grown at high salinity secreted substantial amounts of YwbN in a Tat-independent manner. Most likely, this YwbN secretion takes place via the *B. subtilis* Sec pathway since this pathway has been shown to accept proteins with RR-signal peptides [44]. We attempted to confirm the involvement of Sec in the Tat-independent secretion of YwbN by growing cells in the presence of the SecA inhibitor sodium azide. Unfortunately however, sodium azide interfered significantly with the growth of *B. subtilis* in LB with 6% salt making it impossible to carefully verify a role for the Sec pathway in YwbN secretion. Furthermore, it is presently difficult to say whether the observed Tat-independent secretion of YwbN under high salinity growth conditions relates to indirect effects of these growth conditions on the pre-translocational folding or assembly of YwbN. Since the internal NaCl concentration is unlikely to change in a significant way when cells are grown in LB medium with 6% salt, any possible folding or assembly defects of YwbN could perhaps relate to changes in the intracellular concentrations of as yet unidentified salt stress-dependent proteins, compatible solutes (osmolytes) or ions [45], [46]. Clearly, impaired folding of pre-YwbN would make this protein a potential substrate for the Sec pathway.

The present experiments revealed an essential role of the TatAyCy-secreted YwbN in the acquisition of iron during growth in LB at low salinity and in iron-limiting synthetic BOC medium. At low salinity, the growth defects and post-exponential growth lysis phenomenon of *tatAyCy* or *ywbN* mutants could be fully suppressed by the addition of Fe^2+^, and to a lesser extent Fe^3+^, to the growth medium. This implies that cells lacking TatAyCy and/or YwbN are iron-starved at low salinity. The same turned out to be true for cells lacking the lipoprotein YwbM and the integral membrane protein YwbL. Interestingly, the *E. coli* homologue of YwbM, named EfeO, is a periplasmic protein that has been implicated in high-affinity iron-binding [37]. YwbL is a homologue of the EfeU iron permease in the *E. coli* inner membrane [37], [47], and the high-affinity iron permease Ftr1p of fungi and yeast [48]. While Ftr1p is an Fe^3+^ permease [48], [49], EfeU was shown to permeate Fe^2+^ *in vitro* into proteoliposomes [47]. The present study shows impaired growth of a *ywbL* mutant in the presence of Fe^3+^, but not in the presence of Fe^2+^, indicating that *B. subtilis* YwbL is specific for Fe^3+^ uptake like Ftr1p. Taken together, our findings show that severe iron limitation is the reason why cells lacking YwbLMN or TatAyCy grow at reduced rates in low salinity LB medium, and start to lyse instead of entering the stationary growth phase. The finding that such mutants resume growth after the lysis phase indicates that cell lysis results in the liberation of iron, which can be reused by the surviving cells. This view is confirmed by the observation that the lysis phenomenon can be largely prevented if iron is added to the culture during exponential growth. The precise role of YwbN in making iron available for the cells is not entirely clear but, being a homologue of the Dyp-type iron-dependent peroxidase EfeB, it seems most likely that this enzyme is involved in oxidation of Fe^2+^ for subsequent uptake of Fe^3+^ by YwbL, which is analogous to the yeast Fet3p-Ftr1p high-affinity iron uptake system [27], [37]. Furthermore, recent studies have shown that EfeB of *E. coli* can extract iron from heme without affecting the protoporphyrin ring [50], which suggests a role for *B. subtilis* YwbN in the liberation of complexed iron. A function of YwbN in iron metabolism in *B. subtilis* is fully consistent with the finding by Helmann and co-workers that the *ywbN* gene is part of the Fur regulon [43]. By contrast, *tatAyCy* does not seem to be controlled by Fur, which is consistent with a “mainstream” role in Tat-dependent protein translocation, as documented in the present studies. It is remarkable to note that an iron starvation response was previously documented for *B. subtilis* cells grown at high salinity [51], [52]. This apparent iron limitation at high salinity growth conditions did not result in a requirement for the TatAyCy translocase or YwbLMN as observed under low salinity growth conditions. However, it should be noted that *tatAyCy* mutants do secrete YwbN when grown in LB medium with 6% NaCl, which might suffice for iron acquisition.

Taken together, our present results show for the first time that environmental salinity is a critical determinant for Tat-dependent protein secretion in *B. subtilis.* Depending on the salinity levels, the YwbN protein is seemingly directed from the Tat pathway into the Sec pathway. This observation is reminiscent of our previous observation that the *B. subtilis* esterase LipA can be redirected from the Sec to the Tat pathway under conditions of hyperproduction *via* an overflow mechanism [29]. This suggests that there may be additional, as yet unidentified factors that can impact on the choice between Sec or Tat pathway usage. A parameter that can impact strongly on the rates of protein (un-)folding is temperature. Nevertheless, growth temperature by itself does not seem to be a factor determining secretion pathway dependency, since we did not detect any obvious differences in Tat-dependence of YwbN secretion when cells were grown at 15, 30, 37 or 48°C (unpublished observations). However, being an organism that lives in the soil, *B. subtilis* can be exposed to a plethora of environmental insults that may either directly influence the activity and specificity of the Tat translocases, or that may indirectly impact on the pre-translocational folding of secretory precursor proteins. We are therefore convinced that more proteins will use the Sec and/or Tat pathways of *B. subtilis* in a growth condition-dependent manner. Identification of such conditions will be of interest not only from a fundamental scientific point of view, but also for the biotechnological application of *B. subtilis* as a cell factory for the production of high-value proteins.

## Materials and Methods

### Plasmids, bacterial strains, media and growth conditions

The plasmids and bacterial strains used in this study are listed in Table 1. Strains were grown with agitation at 37°C in Luria Bertani (LB) medium consisting of 1% tryptone, 0.5% yeast extract and 0%, 1% or 6% added NaCl, pH 7.4. Belitsky minimal medium without citrate (BOC medium) was prepared as described [53]. If appropriate, media for were supplemented with the following antibiotics: erythromycin (Em), 2 µg ml^−1^; chloramphenicol (Cm), 5 µg ml^−1^; tetracycline (Tc), 10 µg ml^−1^; spectinomycin (Sp), 100 µg ml^−1^; kanamycin (Km), 20 µg ml^−1^.

### Growth experiments

Strains were pre-cultured in LB medium containing 1% NaCl and subsequently diluted in LB medium without salt or with 1% or 6% NaCl to an optical density at 600 nm (OD_600_) of ∼0.01. Growth was continued in triplicate wells of a 96-well microtiter plate (Greiner) that was incubated in a Biotek Synergy 2 plate reader (37°C, variable shaking). OD_600_ readings were recorded for 8 or 14 hours.

### SDS-PAGE and Western blotting

Cells were separated from the growth medium by centrifugation. Cellular or secreted proteins were separated by SDS-PAGE using pre-cast Bis-Tris NuPAGE gels (Invitrogen). Separated proteins were stained with SYPRO Ruby protein gel stain (Molecular Probes Inc.). The presence of YwbN-Myc, LipA, or TrxA in cellular or growth medium fractions was detected by Western blotting. For this purpose, proteins separated by SDS-PAGE were semi-dry blotted (75 min at 1 mA/Cm^2^) onto a nitrocellulose membrane (Protran®, Schleicher & Schuell). Subsequently, the LipA and TrxA proteins were detected with specific polyclonal antibodies raised in rabbits. YwbN-Myc was detected with monoclonal antibodies against the Myc-tag (Gentaur). Visualisation of bound antibodies was performed with fluorescent IgG secondary antibodies (IRDye 800 CW goat anti-rabbit or goat anti-mouse from LiCor Biosciences) in combination with the Odyssey Infrared Imaging System (LiCor Biosciences). Fluorescence was recorded at 800 nm. Quantification of the recorded data was done using the ImageJ software package (http://rsbweb.nih.gov/ij/).

### Northern blotting analyses

For Northern blotting, cells from overnight cultures in LB medium with 1% NaCl were used to inoculate LB medium without NaCl or with 1% or 6% NaCl. Growth was continued till an OD600 of ∼2 and then the cells were harvested for RNA extraction and blotting. Preparation of total RNA and Northern blot analysis using specific RNA probes were performed as described previously [29]. The RNA preparations (5 or 10 µg per lane) were separated electrophoretically in a 1.2% agarose gel. After hybridization of the blotted RNA with gene-specific probes, chemiluminescence was detected using a Lumi-Imager (Roche Diagnostics). Transcript sizes were determined by comparison with an RNA size marker (Fermentas RiboRuler High Range RNA Ladder). Digoxygenin-labeled specific RNA probes were synthesized by *in vitro* transcription using T7 RNA polymerase and a specific PCR product as template. Synthesis of the DNA template was performed by PCR using the following pairs of oligonucleotides: tatAd-for (5′-ATGTTTTCAAACATTGGAAT-3′) and tatAd-rev (5′-CTAATACGACTCACTATAGGGAGAGCCCGCGTTTTTGTCCTGCT-3′), tatAy-for (5′- ATGCCGATCGGTCCTGGAAG-3′) and tatAy-rev (5′-CTAATACGACTCACTATAGGGAGACTGATCTTCTTTCTTTTTTT-3′). The underlined sequence indicates the T7 promoter region.

## Supporting Information

Figure S1 **TrxA control for cell lysis in media with differing salinity.** Cell and growth medium fractions of *B. subtilis tat* mutant strains and the parental strain 168 were separated by centrifugation and used for SDS-PAGE, Western blotting and immunodetection of the cytoplasmic marker protein TrxA with specific antibodies. The panels show results obtained for cells grown in LB with 1% or 6% NaCl. Protein loading was corrected for OD_600_. The positions of TrxA and Mw markers are indicated. The samples correspond to those of the experiment depicted in Figure 1. No TrxA can be detected in the growth medium fractions indicating that cell lysis was negligible in this experiment.
(TIF)

Figure S2 **Tat-dependence of YwbN secretion in media with differing salinity.** Cell and growth medium fractions of *B. subtilis tat* mutant strains and the parental strain 168 were separated by centrifugation and used for SDS-PAGE and Sypro Ruby staining (left panels) or SDS-PAGE, Western blotting and immunodetection of YwbN-Myc and LipA with specific antibodies (right panels). From top to bottom the panels show results obtained for cells grown in LB with 6%, 1% or no added NaCl. Protein loading was corrected for OD_600_. The YwbN-Myc (YwbN) and LipA proteins, and Mw markers are indicated. A slight 'smiling effect' as observed for the YwbN and LipA bands in the growth medium sample of the total-*tat*2 mutant grown in LB with 6% NaCl is due to some residual salt in the sample (compare also left and right panels). The samples correspond to those of the experiment depicted in Figure 1.
(TIF)

Figure S3 **Growth phenotypes of** ***tatAyCy*** **and** ***ywbLMN*** **mutant strains at low salinity.** *B. subtilis tat* mutant strains or the parental strain 168 were grown for 7.5 to 14 hours in LB medium without NaCl (panels **A-E**), or 1% NaCl (panel **F**). For the experiments in panel **D**, LB medium was supplemented with 10 μM FeCl_3_, and for the experiments in panel **F** with 10 μM FeSO_4_ (panel **E**). **A.** Growth of *tat* mutant strains: *tatAd tatAy* (+), *tatCd* (filled diamonds), *tatCy* (filled triangles), *tatCd tatCy* (filled circles), *tatAdCd* (filled rectangles), *tatAyCy* (open triangles), total-*tat*2 (filled squares). Parental strain 168 (open squares). **B.** Growth of the *tatAyCy* mutant strain complemented with *tatAy* (pCAy; open triangles), *tatCy* (pCCy; X), or *tatAyCy* (pCACy; filled circles). Controls: *tatAyCy* mutant with empty vector pGDL48 (closed squares), parental strain 168 (open squares). **C-F.** Growth of mutant strains: *tatAyCy* (open triangles), *ywbL* (closed diamonds), *ywbM* (X), *ywbN* (open circles), *ywbN* X*ywbN* (no xylose; +), *ywbN* X*ywbN* (plus xylose; closed rectangles). Control: parental strain 168 (open squares).
(TIF)

Figure S4 **Iron additions can prevent the growth defects of** ***tatAyCy*** **and total-** ***tat*** **mutant** ***B. subtilis*** **strains in LB medium without salt.** *B. subtilis tat* mutant strains or the parental strain 168 were grown for 11 hours in LB medium without NaCl. Growth was monitored by OD_600_ readings. The cultures were supplemented with 100 μM FeCl_3_ or 100 μM FeSO_4_ when cells had reached the mid-exponential growth phase after 190 min of cultivation (T1), or when cells had entered the transition phase between the exponential and post-exponential growth phases after 290 min of cultivation (T2). **A.** parental *B. subtilis* strain 168, **B.** *tatAyCy* mutant strain, and **C.** total-*tat* mutant strain. T1 and T2 are marked with arrows. Open squares, no addition to the culture; open triangles, FeCl_3_ was added at T1; filled circles, FeSO_4_ was added at T1; filled squares, FeCl_3_ was added at T2; crosses, FeSO_4_ was added at T2.
(TIF)
