# Supplementary figures and images for: Environmental Salinity Determines the Specificity and Need for Tat-Dependent Secretion of the YwbN Protein in Bacillus subtilis
Source: PLoS One. 2011 Mar 30;6(3):e18140. doi: 10.1371/journal.pone.0018140 (PMC3068169; doi:10.1371/journal.pone.0018140)

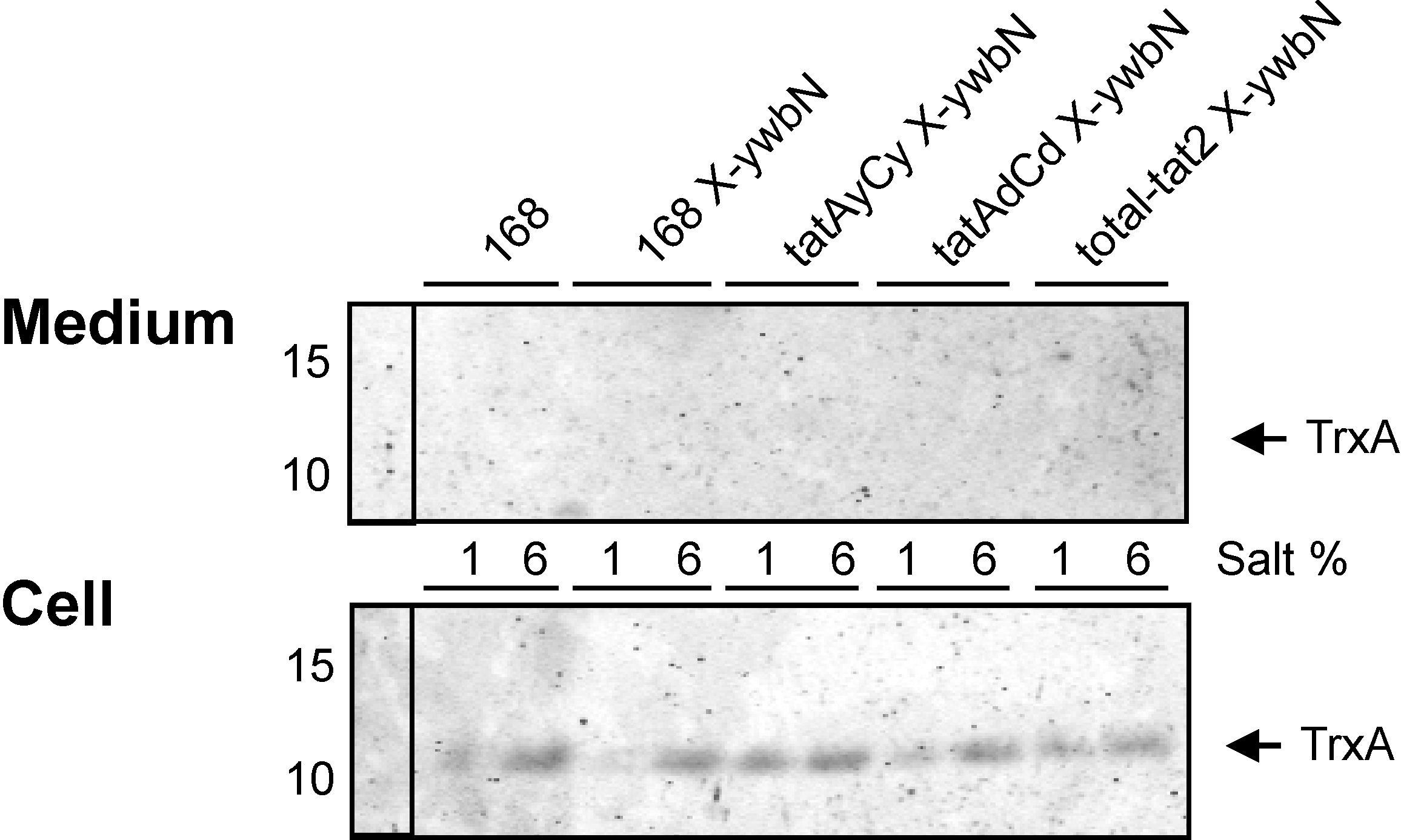

Supplement: Figure S1 — TrxA control for cell lysis in media with differing salinity. Cell and growth medium fractions of B. subtilis tat mutant strains and the parental strain 168 were separated by centrifugation and used for SDS-PAGE, Western blotting and immunodetection of the cytoplasmic marker protein TrxA with specific antibodies. The panels show results obtained for cells grown in LB with 1% or 6% NaCl. Protein loading was corrected for OD600. The positions of TrxA and Mw markers are indicated. The samples correspond to those of the experiment depicted in Figure 1. No TrxA can be detected in the growth medium fractions indicating that cell lysis was negligible in this experiment. (TIF) [file pone.0018140.s001.tif]

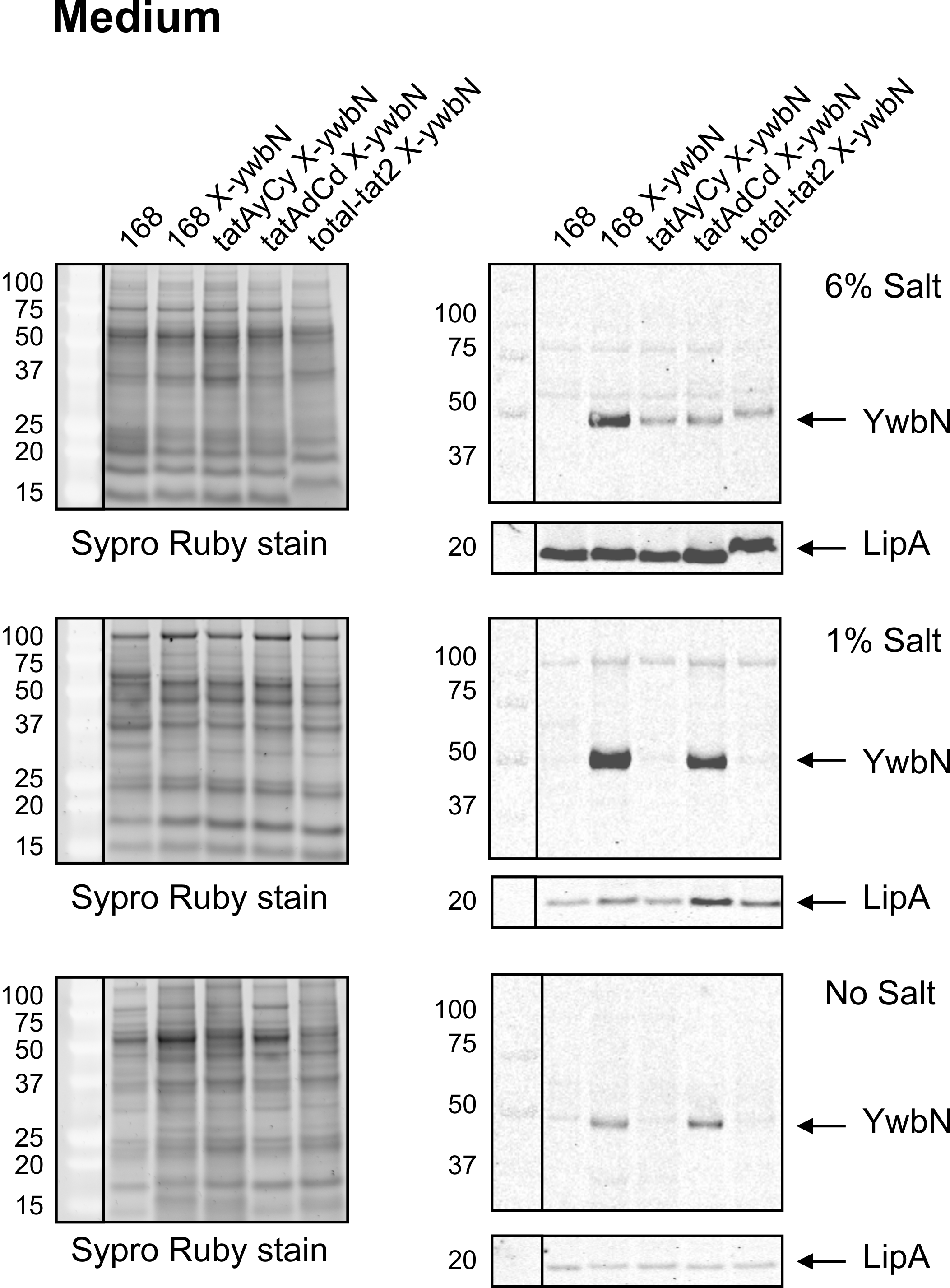

Supplement: Figure S2 — Tat-dependence of YwbN secretion in media with differing salinity. Cell and growth medium fractions of B. subtilis tat mutant strains and the parental strain 168 were separated by centrifugation and used for SDS-PAGE and Sypro Ruby staining (left panels) or SDS-PAGE, Western blotting and immunodetection of YwbN-Myc and LipA with specific antibodies (right panels). From top to bottom the panels show results obtained for cells grown in LB with 6%, 1% or no added NaCl. Protein loading was corrected for OD600. The YwbN-Myc (YwbN) and LipA proteins, and Mw markers are indicated. A slight 'smiling effect' as observed for the YwbN and LipA bands in the growth medium sample of the total-tat2 mutant grown in LB with 6% NaCl is due to some residual salt in the sample (compare also left and right panels). The samples correspond to those of the experiment depicted in Figure 1. (TIF) [file pone.0018140.s002.tif]

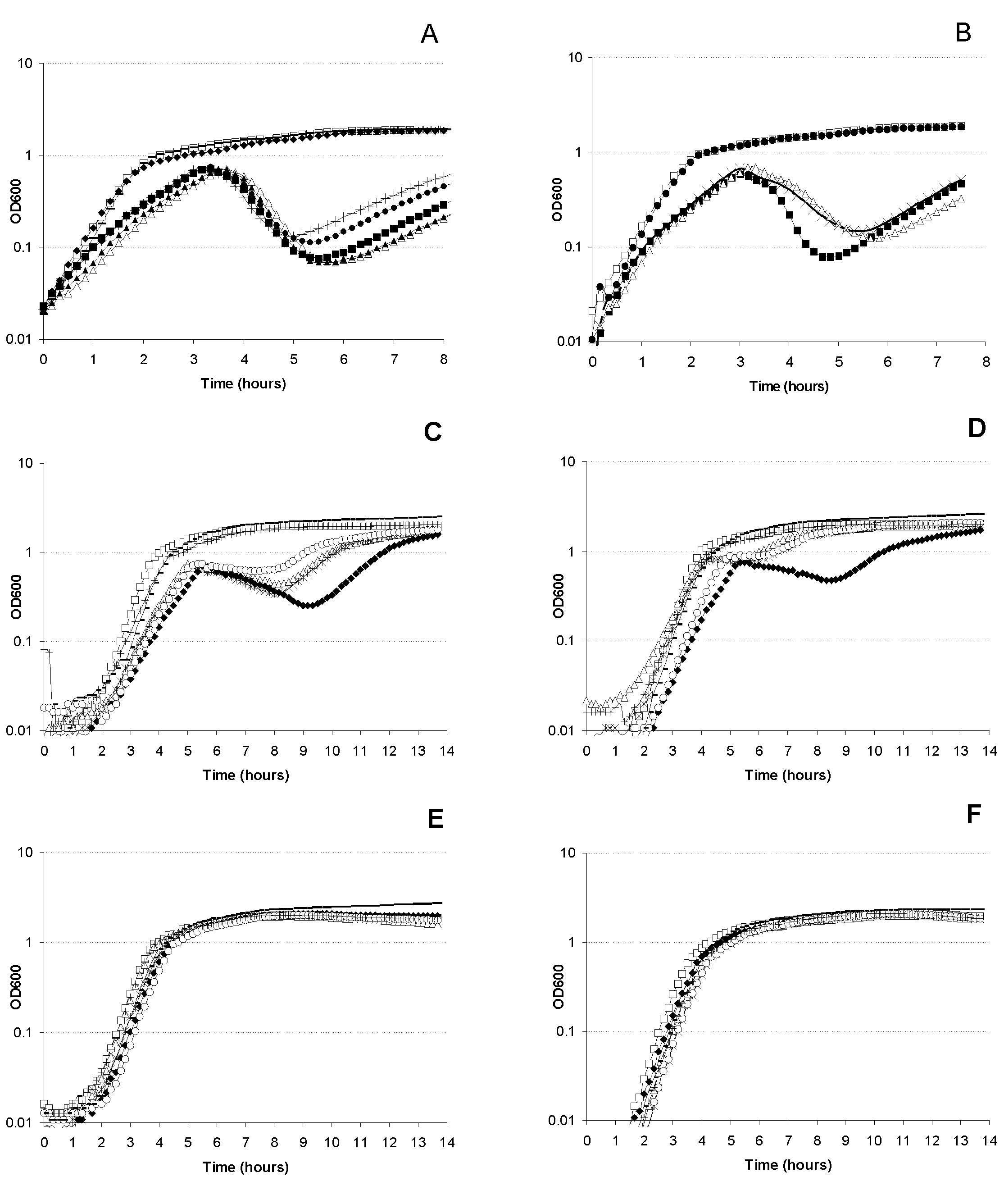

Supplement: Figure S3 — Growth phenotypes of tatAyCy and ywbLMN mutant strains at low salinity. B. subtilis tat mutant strains or the parental strain 168 were grown for 7.5 to 14 hours in LB medium without NaCl (panels A-E), or 1% NaCl (panel F). For the experiments in panel D, LB medium was supplemented with 10 μM FeCl3, and for the experiments in panel F with 10 μM FeSO4 (panel E). A. Growth of tat mutant strains: tatAd tatAy (+), tatCd (filled diamonds), tatCy (filled triangles), tatCd tatCy (filled circles), tatAdCd (filled rectangles), tatAyCy (open triangles), total-tat2 (filled squares). Parental strain 168 (open squares). B. Growth of the tatAyCy mutant strain complemented with tatAy (pCAy; open triangles), tatCy (pCCy; X), or tatAyCy (pCACy; filled circles). Controls: tatAyCy mutant with empty vector pGDL48 (closed squares), parental strain 168 (open squares). C-F. Growth of mutant strains: tatAyCy (open triangles), ywbL (closed diamonds), ywbM (X), ywbN (open circles), ywbN XywbN (no xylose; +), ywbN XywbN (plus xylose; closed rectangles). Control: parental strain 168 (open squares). (TIF) [file pone.0018140.s003.tif]

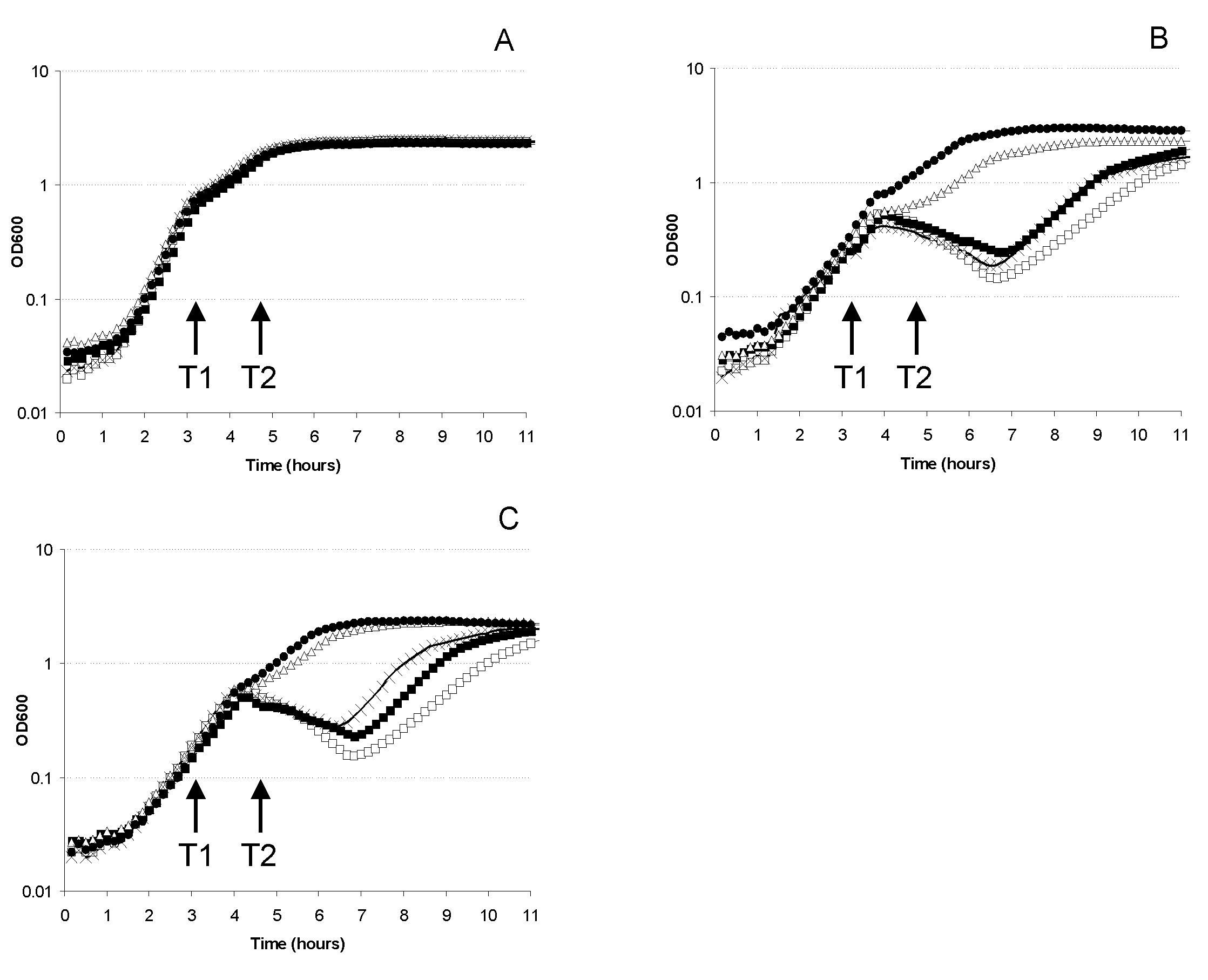

Supplement: Figure S4 — Iron additions can prevent the growth defects of tatAyCy and total- tat mutant B. subtilis strains in LB medium without salt. B. subtilis tat mutant strains or the parental strain 168 were grown for 11 hours in LB medium without NaCl. Growth was monitored by OD600 readings. The cultures were supplemented with 100 μM FeCl3 or 100 μM FeSO4 when cells had reached the mid-exponential growth phase after 190 min of cultivation (T1), or when cells had entered the transition phase between the exponential and post-exponential growth phases after 290 min of cultivation (T2). A. parental B. subtilis strain 168, B. tatAyCy mutant strain, and C. total-tat mutant strain. T1 and T2 are marked with arrows. Open squares, no addition to the culture; open triangles, FeCl3 was added at T1; filled circles, FeSO4 was added at T1; filled squares, FeCl3 was added at T2; crosses, FeSO4 was added at T2. (TIF) [file pone.0018140.s004.tif]
